# QSAR and molecular docking studies on designing potent inhibitors of SARS-CoVs main protease

**DOI:** 10.3389/fphar.2023.1185004

**Published:** 2023-05-05

**Authors:** Fucheng Song, Haoyang Sun, Xiaofang Ma, Wei Wang, Mingyuan Luan, Honglin Zhai, Guanmin Su, Yantao Liu

**Affiliations:** ^1^ Qingdao Municipal Center for Disease Control and Prevention, Qingdao, China; ^2^ Department of Traditional Chinese Medicine, Songshan Hospital of Qingdao University, Qingdao, China; ^3^ Qingdao University Medical College, Qingdao, China; ^4^ Department of Chemistry and Chemical Engineering, Lanzhou University, Lanzhou, China; ^5^ Shandong Provincial Center for Disease Control and Prevention, Jinan, China

**Keywords:** SARS-CoVs, QSAR, unsymmetrical aromatic disulfides, molecular docking, drugs design

## Abstract

**Background:** Severe acute respiratory syndrome coronavirus (SARS-CoVs) have emerged as a global health threat, which had caused a high rate of mortality. There is an urgent need to find effective drugs against these viruses.

**Objective:** This study aims to predict the activity of unsymmetrical aromatic disulfides by constructing a QSAR model, and to design new compounds according to the structural and physicochemical attributes responsible for higher activity towards SARS-CoVs main protease.

**Methods:** All molecules were constructed in ChemOffice software and molecular descriptors were calculated by CODESSA software. A regression-based linear heuristic method was established by changing descriptors datasets and calculating predicted IC_50_ values of compounds. Then, some new compounds were designed according to molecular descriptors from the heuristic method model. The compounds with predicted values smaller than a set point were constantly screened out. Finally, the properties analysis and molecular docking were conducted to further understand the structure-activity relationships of these finalized compounds.

**Results:** The heuristic method explored the various descriptors responsible for bioactivity and gained the best linear model with R^2^ 0.87. The success of the model fully passed the testing set validation, proving that the model has both high statistical significance and excellent predictive ability. A total of 5 compounds with ideal predicted IC_50_ were found from the 96 newly designed derivatives and their properties analyze was carried out. Molecular docking experiments were conducted for the optimal compound 31a, which has the best compound activity with good target protein binding capability.

**Conclusion:** The heuristic method was quite reliable for predicting IC_50_ values of unsymmetrical aromatic disulfides. The present research provides meaningful guidance for further exploration of the highly active inhibitors for SARS-CoVs.

## 1 Introduction

The global epidemic of severe acute respiratory syndrome coronavirus (SARS CoVs) in 2003 had caused a high rate of mortality. Since that, the SARS CoVs have been recognized as a worldwide threat (Konno et al., 2013). SARS CoVs is a large RNA virus of the coronavirus family, which can infect vertebrates, such as humans, pigs, cats and dogs, causing respiratory, gastrointestinal, hepatic, and neurologic diseases (Weiss and Leibowitz, 2011; Delgado-Roche and Mesta, 2020). Symptoms are influenza-like and include high fever, malaise, myalgia, headache, non-productive cough, diarrhea, and shivering (Lee et al., 2003). Due to the recent spread of a new strain from 2019, named SARS-CoV-2, a pathogenic agent of COVID-19 disease, this pathogen has become the center of global attention. To effectively control the spread of the SARS CoVs, many measures have been taken around the world, including control of infection, vaccination, enhanced clinical treatment, and so on. But, the mutant strains of SARS CoVs remain a global pandemic, which can be more dangerous to human health than the previous ones. The re-emergence of new pandemic SARS-CoV-2 has posed a great risk than the strain of the 2003 outbreak (Yang et al., 2020). Therefore it is still an urgent need to design novel anti-SARS CoVs inhibitors to combat this deadly disease.

The study properties of compounds will help to create novel anti-SARS inhibitors with hypo-toxicity, high-bioactivity, and broader protection. We all know that getting a new inhibitor and testing its biological activity and toxicity is a big project that takes a lot of time, energy, and money. Fortunately, powerful artificial intelligence provides practical ways to enter novel structures and foresee security threats. In this regard, quantitative structure-activity relationship (QSAR) is an effective technique for screening novel structures and forecasting a variety of attributes of the synthesized molecules (Saxena et al., 2010; Huang et al., 2011; Jia et al., 2013; Song et al., 2016). The prediction issues of inhibitors action have been settled in the recent 2 decades by creating a bionic mathematics calculation models (Uysal and Tanyildizi, 2012; Reichelt et al., 2014; Song et al., 2017; Si et al., 2021). QSAR model has the potential to make a significant advancement in the development of inhibitors that are both more highly effective and less harmful (Yang et al., 2011; Haudecoeur et al., 2013).

The literature indicates that a variety of QSAR methods has been widely applied in anti-SARS inhibitor researches. Masand et al. (2020) discovered a genetic algorithm-multi-linear regression methodology to modify the peptide-type compounds for SARS-CoV; Zaki et al. (2021) identified anti-SARS-CoV-2 compounds from food by virtual screening, molecular Docking, and molecular dynamics simulation analysis; Galvez-Llompart et al. (2022) described a novel QSAR model based repurposing study on molecular topology and molecular docking for identifying inhibitors of the main protease of SARS-CoV-2; Toropov et al. (2022) obtained new designed anti-SARS CoVs inhibitors based on statistical and probability quality of molecular alerts extracted from SMILES.

Scientific studies show that SARS-CoVs encode a main protease (M^pro^, also called 3CL^pro^) that plays a critical role in processing viral polyproteins and controlling the activity of replicator complexes (Goetz et al., 2007). The M^pro^ at no less than 11 cleavage sites on the large polyprotein 1 ab (replicase 1 ab, ∼790 kDa); the recognition sequence at most sites is LeuGln (Ser, Ala, Gly). Inhibiting the activity of this enzyme would block viral replication the replication of the SARS-CoVs. Generally, the disulfide bonds play essential roles for bioactive proteins to keep correct folding (Zhang et al., 2011). And scientists have long been committed to finding chemical molecules that can control the occurrence of this key node. Since the discovery of unsymmetrical aromatic disulfides possessing novel resistance effects, numerous studies have been conducted to find more powerful anti-M^pro^ inhibitors, including the study of disulfide derivatives and other compounds with similar structural properties. Based on the above two points, the QSAR study was used to analyse and design more protein inhibitors.

The heuristic method (HM) is a wonderful QSAR method to scan all the generated molecular descriptors and analyze the relevant variables with the biological activity (Perestrelo et al., 2019). And the HM models could provide a broader idea for designing new molecule structures. Molecular docking is a contemporary structure-based drug design approach, which provides in-depth knowledge and understanding of binding patterns to identify the important structural features of the enzyme of newly designed molecules (Baig et al., 2016). Therefore, in this study, we employed QSAR-based virtual screening, property analysis, and molecular docking analyses to achieve the desired goals.

## 2 Methods

### 2.1 Data set

Forty unsymmetrical aromatic disulfides derivatives as the inhibitor activities toward SARS CoVs M^pro^ were collected from the literature (Wang et al., 2017). Structures of the studied molecules with their activity IC_50_ values (required concentration of an inhibitor to achieve 50% inhibition of replication of the M^pro^) are illustrated in Table 1. It could be seen that the target compounds exhibited encouraging biological potency, with excellent IC_50_ values ranging from 0.516μM to 5.954 μM. A lower IC_50_ value indicates that has a stronger activity against the M^pro^. The random number method was employed to obtain a distinct set of random numbers. By using this procedure, the subsequent number was unrelated to the preceding one. Finally, 40 compounds are randomly divided into 8 compounds for the testing set and 32 compounds for the training set. The ability and stability of QSAR models created by the training set were assessed by the testing set.

**TABLE 1:** Experimental and predicted IC_50_ of 40 unsymmetrical aromatic disulfides derivatives.

No.	Compound	Exp. (IC_50_)	Pred. (IC_50_)	Residue
1	2-((4-chlorophenyl)disulfanyl)thiazole	1.871	2.120	−0.249
2	N-(2-(p-tolyldisulfanyl)thiazol-5-yl)acetamide	2.803	2.079	0.724
3	Ethyl 2-((4-chlorophenyl)disulfanyl)-1H-imidazole-4-carboxylate	3.657	4.176	−0.519
4^a^	1-(5-Methyl-3-((2-nitrophenyl)disulfanyl)-1H-1,2,4-triazol-1-yl)ethanone	3.130	2.536	0.594
5	N-(2-(phenyldisulfanyl)thiazol-5-yl)acetamide	1.506	1.850	−0.344
6	1-(5-Phenyl-3-(p-tolyldisulfanyl)-1H-1,2,4-triazol-1-yl)ethanone	4.344	4.159	0.185
7^a^	1-(3-((4-methoxyphenyl)disulfanyl)-5-phenyl-1H-1,2,4-triazol-1-yl)ethanone	4.100	4.908	−0.808
8	1-(3-((2-nitrophenyl)disulfanyl)-5-(pyridin-3-yl)-1H-1,2,4-triazol-1-yl)ethanone	1.762	2.443	−0.681
9	Ethyl 2-((1-acetyl-5-(pyridin-3-yl)-1H-1,2,4-triazol-3-yl)disulfanyl)benzoate	5.654	5.202	0.452
10	Ethyl 2-((1-acetyl-5-(pyridin-4-yl)-1H-1,2,4-triazol-3-yl)disulfanyl)benzoate	4.511	4.873	−0.362
11	1-(3-((4-methoxyphenyl)disulfanyl)-5-(pyridin-3-yl)-1H-1,2,4-triazol-1-yl)ethanone	5.794	4.855	0.939
12	N-(2-((4-chlorophenyl)disulfanyl)thiazol-5-yl)acetamide	2.626	2.138	0.488
13	N-(2-((4-bromophenyl)disulfanyl)thiazol-5-yl)acetamide	1.651	2.077	−0.426
14	Methyl 2-((2-nitrophenyl)disulfanyl)-1H-imidazole-4-carboxylate	2.075	2.583	−0.508
15	Methyl 2-((2-(ethoxycarbonyl)phenyl)disulfanyl)-1H-imidazole-4-carboxylate	5.954	5.465	0.489
16	Methyl 2-((2-(methoxycarbonyl)phenyl)disulfanyl)-1H-imidazole-4-carboxylate	3.975	4.435	−0.460
17	Methyl 2-((4-chlorophenyl)disulfanyl)-1H-imidazole-4-carboxylate	4.126	3.611	0.515
18^a^	N-(2-((4-fluorophenyl)disulfanyl)thiazol-5-yl)acetamide	2.565	2.543	0.022
19^a^	N-(2-((2-nitrophenyl)disulfanyl)thiazol-5-yl)acetamide	1.947	1.672	0.275
20	2-((2-nitrophenyl)disulfanyl)thiazole	2.029	1.958	0.071
21^a^	2-(p-tolyldisulfanyl)thiazole	1.250	2.077	−0.827
22^a^	2-((4-fluorophenyl)disulfanyl)thiazole	2.211	1.900	0.311
23	2-((4-bromophenyl)disulfanyl)thiazole	3.321	1.995	1.326
24	4-Methyl-2-((2-nitrophenyl)disulfanyl)thiazole	2.555	2.491	0.064
25	Ethyl 2-((4-methylthiazol-2-yl)disulfanyl)benzoate	2.452	2.982	−0.530
26	Methyl 2-((5-methyl-1,3,4-oxadiazol-2-yl)disulfanyl)benzoate	1.679	1.178	0.501
27^a^	Ethyl 2-((5-methyl-1,3,4-oxadiazol-2-yl)disulfanyl)benzoate	1.557	1.772	−0.215
28	2-Methyl-5-((2-nitrophenyl)disulfanyl)-1,3,4-oxadiazole	1.713	1.578	0.135
29	Methyl 2-((1,3,4-oxadiazol-2-yl)disulfanyl)benzoate	1.118	0.416	0.702
30	Methyl 2-((4-methyloxazol-2-yl)disulfanyl)benzoate	1.264	1.291	−0.027
31	2-((4-chlorophenyl)disulfanyl)-1,3,4-oxadiazole	0.516	0.758	−0.242
32	4,6-Dimethyl-2-((2-nitrophenyl)disulfanyl)pyrimidine	0.921	0.779	0.142
33	2-((4-chlorophenyl)disulfanyl)-4,6-dimethylpyrimidine	1.437	1.708	−0.271
34	2-((4-bromophenyl)disulfanyl)-4,6-dimethylpyrimidine	1.121	1.722	−0.601
35	4,6-Dimethyl-2-(phenyldisulfanyl)pyrimidine	1.991	1.379	0.612
36	4,6-Dimethyl-2-(p-tolyldisulfanyl)pyrimidine	1.495	1.789	−0.294
37^a^	2-((2-nitrophenyl)disulfanyl)pyrimidine	0.833	1.264	−0.431
38	2-((4-chlorophenyl)disulfanyl)pyrimidine	0.864	1.294	−0.430
39	2-((4-bromophenyl)disulfanyl)pyrimidine	0.697	1.237	−0.540
40	2-(p-tolyldisulfanyl)pyrimidine	1.522	1.303	0.219

^a^ The compounds of the testing set. Residue: Exp. (IC_50_)-Pred. (IC_50_).

### 2.2 Calculation of molecular descriptors

Chemical structures are always expressed in a QSAR model as numerical physical and chemical characteristics, which are computed by expert computational chemistry software and are frequently referred to as molecular descriptors. Molecular descriptors are the most crucial parameters in revealing chemical information and influencing the quality of QSAR models. Chemdraw software was used to include all unsymmetrical aromatic disulfides derivatives into molecular structural formulae. First, the MM + molecular mechanics force field was used in the Hyperchem 7.5 application to optimize the molecular structure formulations. The semi-empirical PM3 approach and MOPAC 6.0 software were used to further improve molecular structure formulas in order to provide a more precise optimization (Katritzky et al., 1996). Finally, the calculative process of molecular descriptors was performed by CODESSA software. The molecular descriptors are divided into five groups (Helguera et al., 2008) and include constitutional descriptors, geometrical descriptors, electrostatic descriptors, thermodynamic descriptors, quantum-chemical descriptors, and topological descriptors.

### 2.3 HM linear model

The CODESSA software offers the heuristic approach, a spontaneous linear regression algorithm that can quickly search for a large number of chemical descriptors to provide the best linear equation. The collinearity of the molecular descriptors should be managed during this process. This is not a random mixture of variables. Each descriptor is assessed to guarantee (Si et al., 2011): a) Entire compounds should have a descriptor accessible. The variables that are “0″for the majority of samples are excluded. b) The descriptor values differ from one another and contain more physical and chemical data. c) Any two descriptors’ correlation coefficients should not be higher than 0.8. High correlation variables in the HM model typically lead to inaccurate forecast outcomes. d) F test’s value for the one-parameter correlation with the descriptor is above 1.0. e) The parameter’s t-test value is less than the defined. The coefficient of determination (R^2^), a square of cross-validate coefficient regression (R^2^_CV_), F test value (F), and mean square error (S^2^) were used to assess the stability of the HM model. The following equation can be used in this study to compute the root mean square of HM:

$$RMS=\sqrt{\frac{\sum_{i=1}^{n_{s}}{\left(y_{k}-{\hat{y}}_{k}\right)}^{2}}{n_{s}}}$$

$y_{k}$ : the predictive value, ${\hat{y}}_{k}$ : the experimental value, $n_{s}$ : the number of compounds

Using QSAR method to filtrate and predict the activity of new high-efficient structures has been widely used in drug design. With the help of HM model, changing or adding chemical group to the original structure will help us design new drug molecules purposefully.

### 2.4 Property prediction and molecular docking

In drug discovery, newly designed molecules must meet a set of different criteria. In addition to suitable potency for biological targets, the compound should also be quite selective for unwanted targets and exhibit good physicochemical properties. For instance, charges should be balanced and atom valances not exceeded. Property explorer is a free tool to predict physicochemical and toxicological molecular properties for designing pharmaceutically active compounds (Song et al., 2017). It allows the construction of new molecular structures and property values to be given after automatic analysis. Property includes molecular weight, partition coefficient (LogP), aqueous solubility, topological polar surface area (TPSA), drug-likeness, toxicity assessment and overall drug-score.

Molecular docking has become an indispensable component of the drug discovery process. It enables the identification of new molecules of therapeutic effect, predicting and delineating the interactions of ligand-target at a molecular level. And this approach would assist to design a dosage form in the most cost-effective and time-saving manner. Based on automatic, ligand, and residues three modes, the intended binding site where the molecule can fit can be excavated, and the potential interactions of the molecule with protein can be created by the Sybyl-X 2.0 package (Li et al., 2012).

In this study, molecular docking technology was used to explore the possible interactions of new molecular inhibitors with SARS CoVs M^pro^ at the binding site. Firstly, chemical structures were imported into Sybyl-X software for calculation and optimization. The “max interactions,” “initial optimization,” “max displacement,” “min energy change,” “dielectric constant,” “non-bonded reset,” and “H-bond radius scaling” was set as 1000, 0.01, 0.01, 0.05, 1.0, 10, 0.7, respectively. Then molecules would be assigned Gasteiger-Hückel charges and minimized by the Tripos force field when convergence reached 0.05 kcal/mol/Å. The protein was imported into Sybyl-X software for hydrogenation, charging and optimization. Then, the unnecessary ligands and water molecules were removed. After that, the molecular could bind with protein targets. Finally, the docking results were imported into PyMol software for image optimization. The Amino acid residues and hydrogen bonds were labeled by Pymol software.

## 3 Results

### 3.1 Calculation results of HM

The CODESSA algorithm evaluated 460 descriptors for each chemical in total. To construct the HM model in an organized manner, the various category descriptors would be used as variables. The multi-parameter t-test value for significance was 3.0. The HM model’s evaluation parameters R^2^, R^2^_CV_, S^2^ changed when the number of selected variables varies. It is generally believed that the optimal model can be determined when R^2^_CV_ values decline suddenly or R^2^ values increases less than 0.02 (Votano et al., 2004).

**FIGURE 1:**
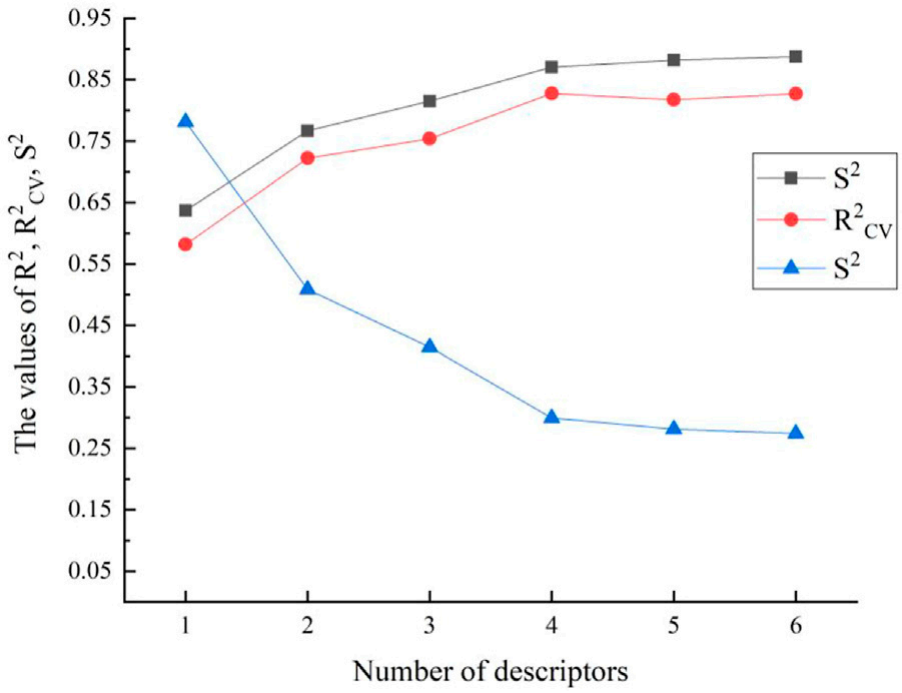
The effects of different number of descriptors on the R^2^, R^2^_CV_, S^2^.

As shown in Figure 1, the best breaking point (R^2^_CV_ declined) occurs when there are four to five variables. At the same time, it also meets the standard that the number of parameters (k) and the sample number (n) require: n ≥ 3 (k + 1). And the F test value for the one-parameter correlation with the descriptor is 65.44. In this way, the HM QSAR model was established, due to the validity ensured by R^2^, F and the stability ensured by R^2^cv. Finally, the 4-variable model should be considered as the optimal linear stable model taking into all the above shreds of evidence. The parameters are WPSA-2 Weighted PPSA (PPSA2*TMSA/1000) (WPSA), Max electroph react index for a C atom (MERIC), Balaban index (BI), and Max coulombic interaction energy for a C-N bond (MCIECNB), Table 2. The t-test value of each parameter is greater than 3.0, which indicates the selected parameters are statistically significant.

**TABLE 2:** The corresponding physicochemical meaning and statistical parameters.

Symbol	Physical–chemical meaning	Non-standardized coefficient	Standardized coefficient	*t*-test
WPSA	WPSA-2 Weighted PPSA (PPSA2*TMSA/1000)	0.0073046	0.77426	12.039
MERIC	Max electroph react index for a C atom	37.795	0.29331	−4.5675
BI	Balaban index	3.8530	0.30594	−4.6424
MCEICNB	Max coulombic interaction energy for a C-N bond	0.65506	0.25369	−3.8547

Table 3 provides the correlation coefficients between the four variables examined by SPSS 20.0 to avoid any multi-collinearity of different variable scales. The correlation coefficient is higher than 0.8, which shows the model is credible and stable. Hence, the HM model can accurately estimate the IC_50_ of unsymmetrical aromatic disulfides derivatives based on statistical data. The following is the linear HM model equation:

$${IC}_{50}=12.825+0.0073046WPSA-37.795MERIC-3.8530BI-0.65506MCIECNB$$

**TABLE 3:** Correlation coefficient of the descriptors in HM model.

	WPSA	MERIC	BI	MCIECNB
WPSA	1.000	0.180	0.238	0.004
MERIC		1.000	0.009	0.232
BI			1.000	0.273
MCIECNB				1.000

**FIGURE 2:**
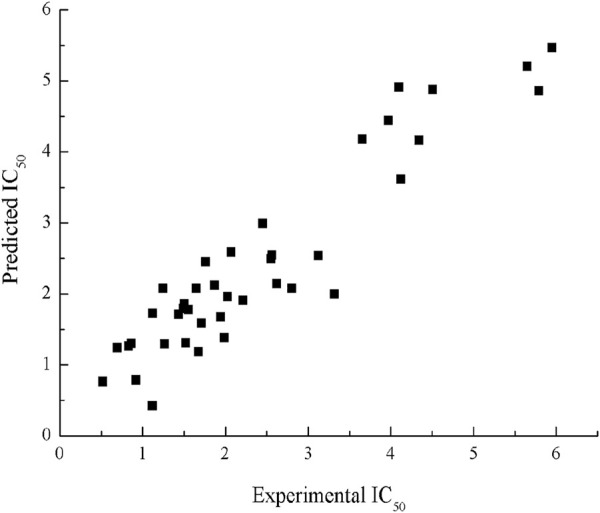
Plot of experimental and predicted IC_50_ by HM.

R^2^cv is 0.82; F = 58.65, *p* < 0.001. The R^2^, S^2^ values for the training set in this model are 0.87 and 0.29, values for the testing set are 0.78 and 0.53, respectively. The scatter plot of experimental and predicted IC_50_ by HM model is shown in Figure 2.

### 3.2 Design of new compounds

The structural factors influencing the IC_50_ values of these compounds might be found by examining the model molecular descriptors. Table 2 provides two different coefficients, the non-standardized coefficient and the standardized coefficient. The non-standardized coefficient is the slope of the regression equation, indicating that each independent variable changes by one amplitude and the dependent variable changes by the corresponding amplitude. Since this coefficient is related to the unit of the independent variable, it is generally not necessary to measure the influence of the independent variable. The standardized coefficient eliminates the influence of independent variable units, and its size can measure the magnitude of the effect of each independent variable on the dependent variable. The larger the absolute value of the standardization coefficient, the greater its impact on the dependent variable (Jonas et al., 2018). According to the absolute values of the standardized coefficient, the contribution of the descriptor to IC_50_ of the compounds is in the order of WPSA > BI > MERIC > MCIECNB. By studying the descriptors included in the HM model, it is possible to obtain some factors related to IC_50_ activity.

WPSA belongs to electrostatic descriptors. Electrostatic descriptors are used to reflect characteristics of the charge distribution of the molecule. This descriptor is based on the Sanderson electronegativity scale and uses the concept which represents molecular electronegativity as a geometric mean of atomic electronegativities. WPSA presents the whole surface area of the molecule and functional group portions and encodes the responsible features for polar interactions between atoms (Luan et al., 2013). In the HM equation, WPSA means effect has the largest positive sign in the model, which suggests that enviable decreased IC_50_ can be achieved by decreasing the ramification of molecular polar interactions.

BI is a very useful molecular topological descriptor with attractive properties. It is an analytical and quantitative method for analyzing the strength of the molecular structure, which is used to measure the relative mechanical stability between atoms or bond energies in substances (Thakur et al., 2004). The size of BI represents the stability of the aromatic ring of organic molecules. The larger it is, the greater the stability of the aromatic ring of organic compounds. As its coefficient in the HM model is negative, with the increasing value of the BI, the IC_50_ value is gradually decreased.

MERIC provides the information about characteristics of the different charge distributions of the C atoms in molecules. MERIC is an electrostatic descriptor that is calculated by using the approach proposed by Zefirov (Si et al., 2007). It can affect the charge of the carbon atoms in the molecule. In this HM model, MERIC has a negative regression coefficient with the IC_50_ values. It suggests that the greater MERIC values, the suppressive ability of SARS CoVs M^pro^ will be enhanced.

MCIECNB is a quantum chemical descriptor. These descriptors characterize the total energy of the molecule in different energy scales and the intramolecular energy distribution using different partitioning schemes (Salman et al., 2006). Max coulombic interaction energy for a C-N bond can reflect the stability of the carbon and nitrogen ring in the unsymmetrical aromatic disulfides derivatives. MCIECNB has a negative sign in the model. This sign suggests that the bigger the coulombic interaction energy between C and N atoms is, the weaker IC_50_, and higher the activity is.

In a conclusion, based on the HM model and interpretation of molecular descriptors, some factors that influenced the inhibitory activity have been found. In this way, the design of new compounds is provided below:

Firstly, the reduction of the polar interactions between atoms of molecules will be extremely favorable to the activity.

Secondly, improvement of the Balaban index of the aromatic ring could enhance the activity.

Thirdly, changing the characteristics of the different charge distributions of the C atoms may be beneficial to improve activity.

Fourthly, to enhance the coulombic interaction energy between C and N atoms.

**FIGURE 3:**
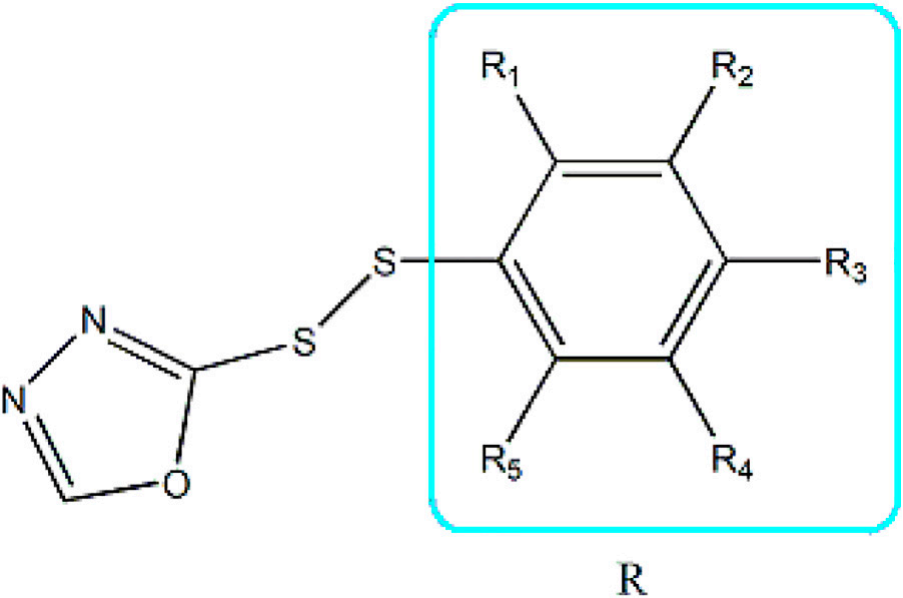
The design strategy mainly focused on the R region of compound 31.

Based on these four factors, the ideal inhibitor structures may be obtained by changing the structural composition of compound 31 (the most potent compound in the literature). The molecular structure adjustments were concentrated in the R region, as shown in Figure 3. There are 6 C atoms in the benzene ring, which is relatively beneficial for the improvement of the Balaban index and the change distribution of different charges. Some Chemical functional groups were introduced and randomly combined at positions R1 to R5 to the reduce the polar interactions between atoms, such as halogen elements, hydroxy, carboxyl, aldehyde group and hydrocarbyl. Furthermore, different forms of C and N atoms have also been involved.

After continuous targeted adjustments and combinations, a total of 96 molecules were designed according to the HM model and descriptors analysis. Then, the physical and chemical parameters of newly designed molecules were calculated by CODESSA software. Entering the physical and chemical parameters of new structures into the HM model will be predicted the IC_50_ values. If the predicted IC_50_ values are lower than compound 31, the corresponding compounds will be taken into property explorer applet analysis and molecular docking study. As a result, only five compounds’ predicted IC_50_ values were lower than 0.516 of compound 31 as shown in Tables 4, 5.

**TABLE 4:** Predicted IC_50_ by HM and analysis results of the property explorer applet of new designed compounds.

No.	Pre.IC_50_	Toxicity	LogP	Solubility	Mol weights	TPSA	Druglikeness	Drug score
31	0.758	Good	2.81	−3.65	244	89.52	0.68	0.69
31a	0.139	Good	1.43	−2.31	251	113.3	0.47	0.75
31b	0.446	Good	2.73	−3.79	302	115.8	−3.46	0.42
31c	0.366	Good	2.81	−3.65	244	89.52	−0.11	0.61
31d	0.212	Good	3.42	−4.39	278	89.52	0.45	0.59
31e	0.449	Good	2.41	−3.55	246	89.52	−2.93	0.45

**TABLE 5:** Docking total score of new design compounds.

No.	Compounds	Total score
31	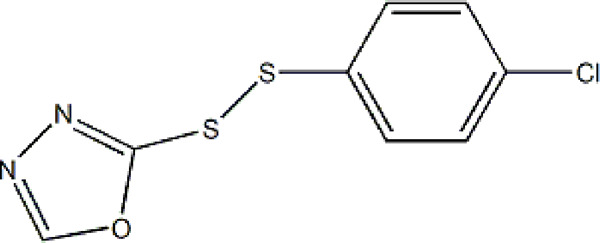	3.203
31a	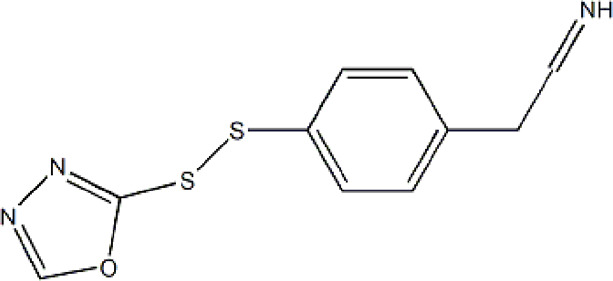	4.564
31b	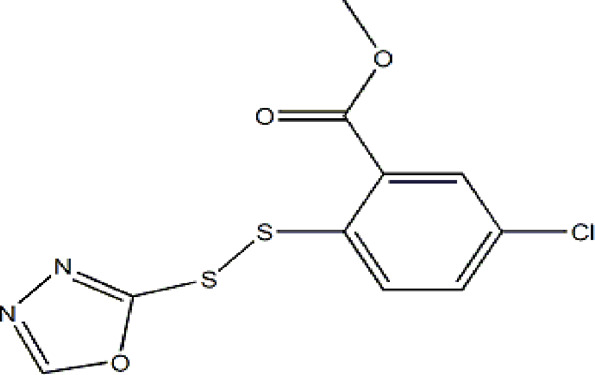	4.078
31c	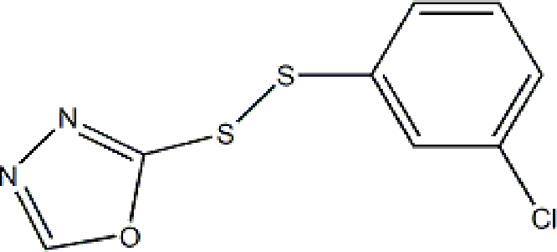	3.271
31d	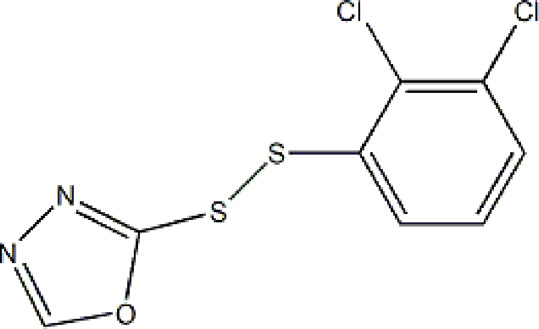	1.914
31e	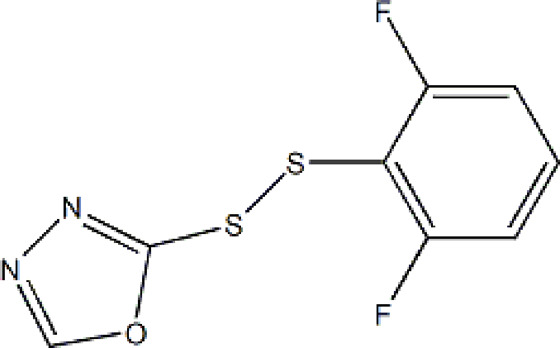	1.837

### 3.3 Property prediction of new compounds

In the Property explorer applet, the prediction results are presented digitally and color-coded. Toxicity assessment includes mutagenic, tumorigenic, irritant, and reproductive effects. The prediction process relies on a pre-computed set of the structural fragment that gives rise to toxicity alerts in case they are encountered in the structure currently drawn. The green signal indicates the compounds are largely free of toxic effects. When there is a red signal, it means the compounds most likely have a high risk of toxicology effects. The logP value is the logarithm of its partition coefficient between n-octanol and water, which is a well-established measure of the compound’s hydrophilicity. It has been shown that for compounds with a reasonable threshold of good absorption, the logP value must be no greater than 5.0. The aqueous solubility of compounds significantly affects their intestinal absorption and cellular distribution characteristics. Usually, lower solubility is accompanied by poor absorption, so the general goal is to get higher solubility of compounds. TPSA is the total sum of all topological polar regions of a molecule’s surface, which correlates well with various bioavailability-related properties, such as blood-brain barrier penetration (Ertl et al., 2000). If the contribution of TPSA is greater, then the molecules’ likelihood to pass membranes easily is significantly reduced. Molecular weights influence the biological activity of compounds. Compounds with lower weights are more likely to be absorbed and distributed. So, Thus, reducing molecular weights as much as possible should be the desire of every drug counterfeiter. Druglikeness is applied in new drug design to evaluate how it ‘druglike’ an object that is regarding factors, such as bioavailability (Smith, 2011). About 80% of the drugs have a positive druglikeness value whereas the big majority of commercially available chemicals account for the negative values. Thus, it is important to keep newly designed compounds in the positive range.

The drug score is an eventual criterion used to synthetically judge the compound’s overall potential to qualify for a drug. It is calculated by the combining of the above property parameters. All the drug scores about the newly designed compounds are listed in Table 5.

### 3.4 Molecular docking of new compounds

**FIGURE 4:**
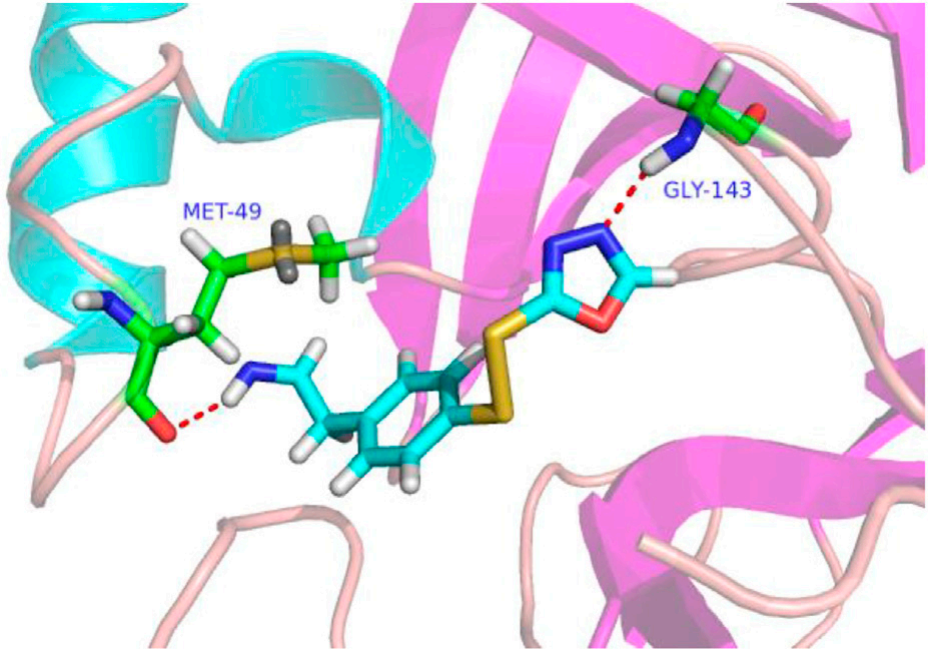
Docking assay of compound 31a with SARS-CoVs M^pro^ related target (PDB ID: 2AMD). In the images of residues Met-49 and GLY-143, carbon atoms are shown in green, oxygen atoms in red, nitrogen atoms in blue, sulfur atoms in yellow, hydrogen atom in white. The red dash lines show the bonding reaction between compound 31a and residues.

In molecular docking experiments, the new compounds were used as ligands for docking with the SARS-CoVs M^pro^ (pdb code 2AMD). As a result, compound 31a had the highest molecular docking total score 4.564, which was a lot higher than compound 31. Figure 4 shows a detailed binding mode with two hydrogen bond interactions formed with residues.

From the docking conformation of compound 31a, the nitrogen atom (located in the structure 1,3,4-oxadiazole) formed a hydrogen bond with the residue GLY-143, this is the same binding mode as that of compound 31 (Wang et al., 2017). And the nitrogen atom of the newly designed structural component formed a hydrogen bond with MET-49. It appears that compound 31a can form a strong bonding reaction with SARS CoVs M^pro^, and maybe a promising candidate inhibitor for this protease.

## 4 Conclusion

In this study, the linear QSAR approaches HM was successfully used to demonstrate the relationship between unsymmetrical aromatic disulfides derivatives with SARS CoVs M^pro^ experimentally, and it can be seen from the experimental results that the descriptor WPSA has the greatest influence on compound activity. Finally, 96 new derivatives were designed according to the molecular descriptors and suggest some new compounds with possible great activities. Based on the property explorer applet, compound 31a passed the stringent criteria and got the highest drug score. To further verify the biological activity, molecule docking experiments were performed and indicating that compound 31a also formed a strong bonding reaction with SARS CoVs M^pro^. In conclusion, these results could provide new instructions for further exploration of the highly active inhibitors for SARS CoVs.

## Data Availability

The original contributions presented in the study are included in the article/supplementary materials, further inquiries can be directed to the corresponding authors.
